# Acute Coronary Syndrome After Aneurysmal Subarachnoid Hemorrhage: Incidence, Risk Factors and Impact on the Outcome

**DOI:** 10.3390/medicina60111862

**Published:** 2024-11-14

**Authors:** Džiugas Meška, Sebastian Schroer, Svenja Odensass, Meltem Gümüs, Christoph Rieß, Thiemo F. Dinger, Laurèl Rauschenbach, Adrian Engel, Marvin Darkwah Oppong, Yahya Ahmadipour, Yan Li, Philipp Dammann, Ulrich Sure, Ramazan Jabbarli

**Affiliations:** 1 Department of Neurosurgery and Spine Surgery, University Hospital Essen, University of Duisburg-Essen, 45147 Essen, Germany; sebastian.schroer@uk-essen.de (S.S.); svenja.odensass@uk-essen.de (S.O.); meltem.guemues@uk-essen.de (M.G.); christoph.riess@uk-essen.de (C.R.); thiemo-florin.dinger@uk-essen.de (T.F.D.); laurel.rauschenbach@uk-essen.de (L.R.); adrian.engel@uk-essen.de (A.E.); marvin.darkwahoppong@uk-essen.de (M.D.O.); yahya.ahmadipour@uk-essen.de (Y.A.); philipp.dammann@uk-essen.de (P.D.); ulrich.sure@uk-essen.de (U.S.); ramazan.jabbarli@uk-essen.de (R.J.); 2 Department of Diagnostic and Interventional Radiology and Neuroradiology, University Hospital Essen, University of Duisburg-Essen, 45147 Essen, Germany; yan.li@uk-essen.de

**Keywords:** aneurysmal subarachnoid hemorrhage, acute coronary syndrome, incidence, risk factors, outcome, score, early prognosis, critical care

## Abstract

*Background and Objectives:* Development of acute coronary syndrome (ACS) after aneurysmal subarachnoid hemorrhage (aSAH) strongly affects further neuro-intensive care management. We aimed to analyze the incidence, risk factors and clinical impact of ACS in aSAH patients. *Materials and Methods:* This retrospective analysis included 855 aSAH cases treated between 01/2003 and 06/2016. The occurrence of ACS during 3 weeks of aSAH was documented. Patients’ demographic, clinical, radiographic and laboratory characteristics at admission were collected as potential ACS predictors. The association between ACS and the aSAH outcome was analyzed as the occurrence of cerebral infarcts in the computed tomography scans and unfavorable outcome (modified Rankin scale > 3) at 6 months after aSAH. Univariable and multivariable analyses were performed. *Results:* ACS was documented in 28 cases (3.3%) in the final cohort (mean age: 54.9 years; 67.8% females). In the multivariable analysis, there was a significant association between ACS, an unfavorable outcome (adjusted odds ratio [aOR] = 3.43, *p* = 0.027) and a borderline significance with cerebral infarcts (aOR = 2.5, *p* = 0.066). The final prediction model for ACS occurrence included five independent predictors (age > 55 years [1 point], serum sodium < 142 mmol/L [3 points], blood sugar ≥ 170 mg/dL [2 points], serum creatine kinase ≥ 255 U/L [3 points] and gamma-glutamyl transferase ≥ 36 U/L [1 point]) and showed high diagnostic accuracy for ACS prediction (AUC = 0.879). Depending on the cumulative score value, the risk of ACS in the cohort varied between 0% (0 points) and 66.7% (10 points). *Conclusions:* ACS is a rare, but clinically very relevant, complication of aSAH. The development of ACS can reliably be predicted by the presented prediction model, which enables the early identification of aSAH individuals at high risk for ACS. External validation of the prediction model is mandatory.

## 1. Introduction

Aneurysmal subarachnoid hemorrhage (aSAH) accounts for approximately 5% of all strokes. Because it occurs at a young age and has a high case fatality, the loss of productive life years in the general population from aSAH is as large as that from ischemic stroke [1]. Important risk factors are familial predisposition, hypertension, smoking and alcohol abuse [2]. The global incidence of aneurysmal aSAH is 7.9 per 100,000 person-years [3]. Various complications of aSAH lead to further deterioration of the neurological outcome and even death. A distinction is made between neurological and extracerebral complications. The most common neurological complications are vasospasm or delayed cerebral ischemia (DCI) as well as increased intracranial pressure (ICP) or hydrocephalus [4]. The relevant extracerebral complications are pneumonia, sepsis and septic shock, as well as acute coronary syndrome (ACS).

ACS in aSAH patients was reported in the literature as early as the 1990s with various case reports [5,6,7]. Later, connections between electrocardiography (ECG) changes typical of coronary syndromes and morbidity and mortality in aSAH patients were postulated [8,9,10]. The concordance of both aSAH and ACS sometimes had fatal consequences for the patients [11]. In recent publications, there has now been an increasing search for the pathomechanism of this phenomenon. It has been suspected that neurogenic stress and endogenous catecholamine excess cause transient cardiomyopathy, leading to laboratory and ECG changes typical of myocardial infarction [12,13].

An additional crucial consideration lies in the interplay between the normal functioning of the cardiac system and the incidence of ACS in shaping the trajectory and prognosis of SAH. In a prospective multicenter cohort study, echocardiographically measured midventricular wall motion abnormalities were independently associated with the risk of DCI, death, and a poor outcome, whereas elevated troponin T levels, ST-segment changes, and low voltage on the admission ECG were not related to aSAH outcomes [14]. In another study, cardiac enzymes, heart rate and systolic blood pressure were significantly associated with death [15]. Lastly, cardiac complications significantly impacted the outcomes of aSAH patients up to 6 months following hemorrhage were shown in a recent publication [16].

Despite the clinical relevance of the ACS for aSAH patients, there is still no tool for the cumulative assessment of the risk of ACS after aSAH. Therefore, we aimed to develop a risk score for this specific group of patients that could help to predict the occurrence of ACS during aSAH upon the characteristics available at disease onset.

## 2. Materials and Methods

### 2.1. Patient Population

Data for this study comes from the long-term observation study ARCTICA (Assessment of Risk Clusters for Treatment of Individuals with Cerebral Aneurysms) at the University Hospital Essen. This database collected clinical, demographic and radiological parameters of patients with intracranial aneurysms between January 2003 and June 2016.

In this period, 995 consecutive aSAH cases were treated at our institution. The prerequisite criteria for inclusion were the image morphological or clinical evidence of an acute aSAH with an aneurysmal origin of bleeding, determined by digital subtraction angiography (DSA) or computed tomography (CT) angiography, as well as the availability of intensive care monitoring records for at least 14 consecutive days after the bleeding event. Cases with shorter survival were excluded, and none of the excluded cases exhibited clinical, ECG or biochemical markers of ACS. Patients with delayed hospital admission (≥24 h post-aSAH), earlier transferal to other hospitals (≤14 days post-aSAH), or the lack of required intensive care records and/or routine admission laboratory tests were excluded from this study (*n* = 140, see Supplementary Figure S1). Accordingly, 855 cases with aSAH were included in the final cohort.

The approval of the institutional ethics committee (Ethik-Kommission der Medizinischen Fakultät der Universität Duisburg-Essen) for this study was obtained (registration number: 15-6331-BO, Approval Date: 01/02/2016). This study has been performed in accordance with the ethical standards laid down in the 1964 Declaration of Helsinki and its later amendments. All patients or their relatives gave written informed consent within the treatment contract before inclusion into the database. This study is also included in the German Register of Clinical Studies (DRKSs) under number DRKS00008749.

### 2.2. Treatment Course of aSAH Patients

At the University Hospital Essen, patients with suspected or confirmed aSAH were admitted via the emergency department. At this point, the clinical neurological assessment was carried out, and the patients were classified according to the World Federation of Neurological Surgeon (WFNS) scales. In addition, laboratory values such as blood count, clinical chemistry and plasma coagulation parameters were routinely taken. All patients with a suspected aSAH underwent DSA or CT angiography of the head to identify the bleeding source. Aneurysm treatment was performed within 24 h after hospital admission by endovascular coiling or microsurgical clipping. Acute hydrocephalus was treated by the insertion of an external ventricular drain allowing the measurement of the ICP. aSAH patients were treated in the neurosurgical intensive care unit, where further care was provided in accordance with the latest international treatment guidelines [17,18,19] for at least 14 days including a 24 h ECG, pulse oximetry and invasive blood pressure monitoring. Normoglycemia was aimed for and maintained. Moreover, transcranial Doppler examinations were carried out daily to detect vasospasms at an early stage. Preventively, patients received the calcium channel antagonist nimodipine for 21 days [20]. Vasospasm was treated interventionally by intra-arterial nimodipine, or intraluminal angioplasty. A raised ICP of >20 mmHg was initially treated conservatively by the drainage of cerebrospinal fluid, head elevation, osmotherapy, deep sedation and relaxation. Patients with increased ICP refractory to conservative management underwent decompressive craniectomy.

### 2.3. ACS Diagnosis and Management in aSAH Patients

In cases of suspected ACS upon 24 h ECG monitoring and/or clinical symptoms (such as chest pain and cardiocirculatory instability), further diagnosis was established, including a 12-channel ECG, measuring the cardiac enzymes, namely, Troponin, creatine kinase (CK) and creatine kinase—myocardial band (CK-MB), as well as obtaining a transthoracic echocardiography. Criteria to diagnose ST-elevation myocardial infarction (STEMI) in an ECG included the ST segment elevation of 2 mm in men and 1.5 mm in women for leads V2 and V3; 1 mm for leads V1, V4–6, I, II, III, aVL and aVF, as well as the presence of a new left bundle branch block in the setting of chest pain. Criteria to diagnose STEMI in cardiac biomarkers was a Troponin I value above the 99th percentile of the healthy population (in our clinical chemistry laboratory defined as above 45 ng/L), elevated levels of CK and CK-MB had only supportive values for diagnosis. The confirmation of diagnosis and management of ACS was performed interdisciplinary with the cardiology department of the university hospital, depending on the severity of both the aSAH and the hemodynamic relevance of the ACS. Supportive therapy includes calculated oxygen supply, or the administration of cardioprotective medications [21].

### 2.4. Data Management

The variables of interest, including demographic characteristics (age, sex and ethnicity) of the patients and their previous medical history (comorbidities, regular medication), radiographic and clinical severity of aSAH, initial laboratory and vital parameters were collected from the electronic patients’ records and the institutional retrospective aneurysm database. The initial clinical severity of aSAH was classified using the WFNS scale [22] and was dichotomized into good (WFNS 1–3) and poor (WFNS 4–5) grades for statistical analysis. The original Fisher scale was used to assess the radiological severity of bleeding [23], with further dichotomization into high (Fisher 3–4) and low (Fisher 1–2) grades. Other characteristics of aSAH, such as the presence of intracerebral and intraventricular hemorrhage, the severity of intraventricular hemorrhage according to the original Graeb score [24], the severity of aSAH according to the Hijdra sum score [25], aneurysm location and size and treatment modality were also analyzed. Lastly, the relative risk of ACS concerning the major clinical events (persistent ICP elevation, decompressive craniectomy, presence and treatment of cerebral vasospasm) during aSAH treatment course was evaluated.

### 2.5. Statistical Analysis

The main goal of this study was to evaluate the ability to predict an ACS event upon the variables available at the moment of patients’ admission. Therefore, the primary endpoint was the construction of a risk score that would enable the stratification of aSAH patients for ACS risk as early as within 24 h after ictus. The secondary endpoints of this study were the influence of ACS on in-hospital mortality, the development of new cerebral infarctions and a long-term functional outcome at 6 months after aSAH, defined as the modified Rankin scale (mRS) > 3 [26].

First, univariate analyses were performed. Age was dichotomized at the cohorts’ mean age. The remaining continuous variables were first tested using the Mann–Whitney U test for non-normally distributed data and the t-test for normally distributed data. Then, continuous variables with associations reaching the *p*-value of <0.1 were subsequently dichotomized at a clinically relevant cutoff using the receiver operating characteristic (ROC) curve analysis. The categorical variables were reported with relative or absolute frequencies and percentages and were analyzed using the chi-square test. If the sample sizes were smaller than 5, the Fisher exact test was used.

Finally, the associations with a *p*-value of <0.1 were included in a backward multivariate regression analysis to identify independent associations and to develop a scoring system for cumulative risk assessment. To weigh the individual predictors of the risk score, the respective adjusted odds ratio (aOR) of the individual independent predictors was divided by the smallest aOR of all values. The results were rounded to the nearest whole number, assigning weights to the individual predictors in the scoring system. The risk score’s diagnostic value for predicting ACS was then analyzed using an ROC curve. The area under the curve (AUC) demonstrated the prediction quality.

The associations between ACS and aSAH outcome parameters were adjusted in the final multivariate analysis for common aSAH confounders (radiological and clinical severity of aSAH, patients’ age, and the type of aneurysm occlusion). The statistical analysis was performed using the SPSS 27 software for Windows (IBM Corp., Armonk, NY, USA). The significance level was set at α = 0.05. The missing values were replaced using multiple imputations.

## 3. Results

The mean age of 855 aSAH patients in the final cohort was 54.9 years (±13.83) and 667 of them (67%) were female. Sixty-one percent of patients were treated with microsurgical clipping. ACS was documented in 28 cases (3.3%). The in-hospital mortality rate was 17.7% (*n* = 151), and the unfavorable outcome after 6 months was observed in 297 patients (37.3%). More detailed information on the baseline characteristics of the aSAH cohort is provided in Table 1.

### 3.1. Factors Associated with ACS and Risk Score Construction

From the univariate analysis (Table 2, see also Supplementary Table S1 with the primary evaluation of continuous variables), higher age (>55 years, odds ratio [OR] = 2.66, *p* = 0.017), presence (OR = 2.09, *p* = 0.077) and severity of intraventricular hemorrhage (Graeb Score ≥ 5 points, OR = 2.57, *p* = 0.025), poor initial clinical condition (WFNS grade = 4–5, OR = 2.48, *p* = 0.028), white blood cell count (≥11.0 × 109/L, OR = 2.81, *p* = 0.03), maximal body temperature (>38.0°, OR = 2.43, *p* = 0.038), sodium (<142 mmol/L, OR = 11.44, *p* = 0.001), glucose (≥170 mg/dL, OR = 6.56, *p* < 0.0001), urea (<17 mg/dL, OR = 0.46, *p* = 0.091), creatinine kinase (CK ≥ 255 U/L, OR = 5.85, *p* < 0.0001), Glutamate-Oxaloacetate-Transaminase (GOT ≥ 30 U/L, OR = 4.29, *p* = 0.001), Glutamate-Pyruvate-Transaminase (GPT ≥ 26 U/L, OR = 2.99, *p* = 0.015), Gamma-Glutamyl transferase (GGT ≥ 36 U/L, OR = 3.89, *p* = 0.003) and Lactate Dehydrogenase (LDH ≥ 260 U/L, OR = 4.99, *p* < 0.0001) levels in blood at admission were selected for further statistical evaluation. Pre-existing cardiac disease was not associated with ACS (OR = 1.57, *p* = 0.342).

In the final step of the multivariable backward regression analysis (Table 3), the following parameters remained significantly associated with ACS after ten regression steps: age > 55 years (aOR = 3.36, *p* = 0.040), sodium < 142 mmol/L (aOR = 8.99, *p* = 0.040), glucose ≥ 170 mg/dL (aOR = 5.01, *p* = 0.004), CK ≥ 255 U/L (aOR = 10.79, *p* < 0.0001) and GGT ≥ 36 U/L (aOR = 3.19, *p* = 0.042, see also Supplementary Table S2 for all ten steps of the backward regression analysis). These five independent ACS predictors were then included in a novel risk score. As both serum sodium < 142 mmol/L and CK ≥ 255 U/L had the greatest aOR values, they each received three points in the weighting. The next predictor, glucose ≥ 170 mg/dL, was weighted with two points, whereas age > 55 years and GGT ≥ 36 U/L—by one point each. Accordingly, the constructed ACS risk score ranged between 0 (minimum) and 10 (maximum) points.

**Figure 1:**
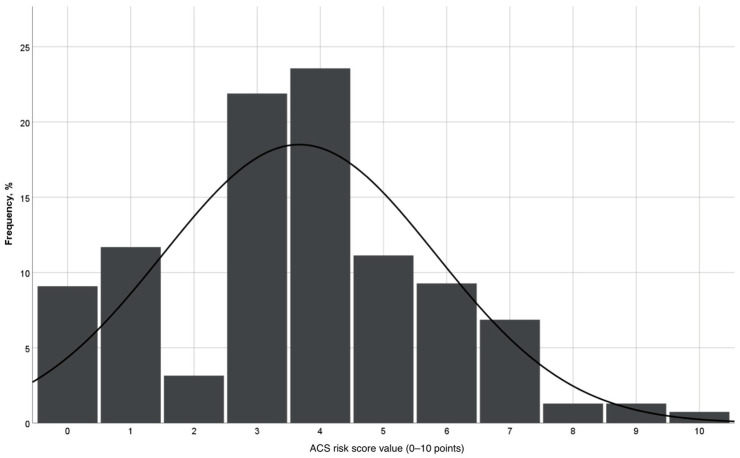
Histogram representing ACS risk score value distribution in the study population.

**Figure 2:**
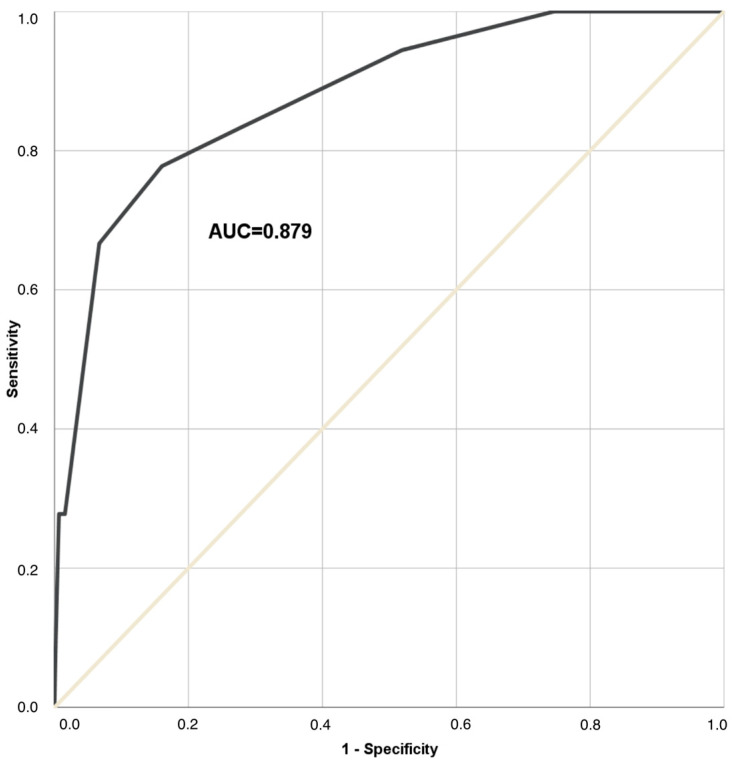
ROC curve showing a good diagnostic accuracy of the novel risk score for ACS prediction, with the area under the curve of 0.897 (*p* < 0.0001).

**Figure 3:**
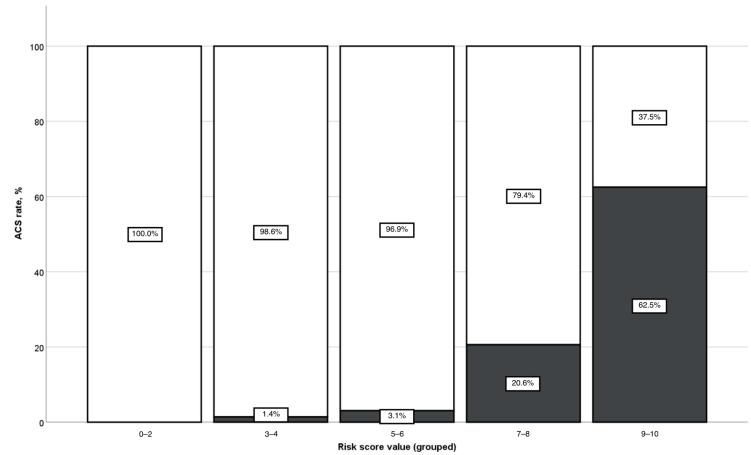
Bar chart showing the rates of ACS (gray bars) in the study population depending on the value of the ACS risk score.

The risk score was then calculated for all patients in the cohort. The mean risk score value in the analyzed aSAH population was 3.67 points (±2.16). Figure 1 demonstrates the distribution of ACS score values in the study population. The diagnostic accuracy of the constructed risk score for the ACS prediction was tested using the ROC analysis (Figure 2). Here, we could show a high predictive power for the novel risk score for ACS events, with an AUC of 0.879 (*p* < 0.001). Figure 3 demonstrates the increase in risk with an increasing ACS prediction score. In particular, none of aSAH patients scoring < 2 points developed ACS. In contrast, for aSAH patients with a score ≥ 9 points, the relative risk of ACS was 62.5%.

### 3.2. Relative Risk of ACS in Major Clinical aSAH Events

None of the major clinical events in aSAH treatment had a significant effect on the incidence of ACS (see Supplementary Table S3 in Online Supplements). ACS occurred in 4.3% of patients who underwent decompressive craniectomy compared to 3.1% in those who did not. The difference was not significant (OR = 1.41, *p* = 0.47). Persistent ICP elevation (OR = 1.16, *p* = 0.69) and the occurrence of symptomatic cerebral vasospasms (OR = 0.92, *p* = 1.000) were also not associated with the increased risk of ACS.

### 3.3. ACS Event and aSAH Outcome Parameters

The incidence of unfavorable outcomes after 6 months among patients who suffered ACS was 66.7% (16 of 24 cases with an available 6-month outcome), which was significantly higher than the rate in patients who had no ACS event—36.4% (281 of 773 patients). A multivariable analysis (Table 4) confirmed an independent association between ACS and an unfavorable functional outcome at 6 months after aSAH: aOR 3.43, *p* = 0.027. The rate of cerebral infarction was also higher in aSAH individuals with ACS (19 of 27 cases, 70%) than in counterparts without ACS (392 of 824 cases, 47.6%). However, multivariable analysis showed only a borderline significance for the association between ACS and cerebral infarcts in follow-up CT scans: aOR = aOR 2.49, *p* = 0.066. Finally, there was a non-significantly higher rate of in-hospital mortality among ACS patients (7/28 [25%] vs. 144/827 [17.4%] in the non-ACS subgroup), with no association in the multivariable analysis (aOR = 1.13, *p* = 0.825).

## 4. Discussion

In the current literature, connections have been established between disease-typical parameters of ACS, such as Troponin-I or N-terminal pro–B-type natriuretic peptide (NT-proBNP) elevations, and poor neurological outcomes after aSAH [27,28], but an early identification of patients at risk has not yet been possible.

The aim of this study was to re-evaluate risk factors available at admission in order to construct a risk score for the early prediction of ACS in aSAH patients. The independent predictors identified in this study, age and routine laboratory parameters were finally combined into a novel risk score, which showed a remarkable diagnostic accuracy in the analyzed aSAH population.

We identified age as an independent predictor of ACS during aSAH. Senior patient age is also considered a risk factor for coronary heart disease or myocardial infarction in studies independent of aSAH [29]. The available data in aSAH patients is congruent [30]. With a cut-off value for age (>55), we were able to select the first independent significant predictor for this score.

The other four predictors originate in clinical chemistry. This trait is a novelty due to the fact that, in other studies, expensive specific cardiac markers were obligatory to establish the connection between cardiac events in aSAH patients and their outcome relevance [27,28]. Our predictors have the advantage of being a part of the routine laboratory evaluation upon admission. Therefore, by using this risk score, the patients can be identified as early as possible, and, most importantly, before myocardial damage. If the cardiac markers, such as cardiac enzymes from the aforementioned studies, are elevated, damage has already occurred.

In our study, admission glucose with a cut-off value (≥170 mg/dL) independently predicted ACS after aSAH. Hyperglycemia on admission, irrespective of whether the patient is diabetic, is an independent predictor of cardiac outcome, including congestive heart failure, cardiogenic shock, and death after acute myocardial infarction [31]. Moreover, acute myocardial infarction patients without diabetes who have stress hyperglycemia on admission are at increased risk of in-hospital mortality and congestive heart failure or cardiogenic shock [32]. The association between admission blood glucose levels and patients’ outcomes has also been observed in ischemic and hemorrhagic stroke, including aSAH [33]. In particular, a retrospective study including 417 stroke patients found that it is admission hyperglycemia, rather than diabetes mellitus, that is a predictor of poor functional status in patients treated with thrombectomy [34]. Another retrospective analysis of 1376 stroke patients suggested that stress hyperglycemia, defined by the glucose/HbA1c ratio, is associated with increased short-term and long-term mortality in patients with ischemic stroke, independent of the patient’s diabetes status [35]. Lastly, a sub-study of a prospective observational cohort study including 987 sepsis patients, reported on an association between hyperglycemia at admission with an adverse outcome of sepsis irrespective of the presence or absence of preexisting diabetes, by a mechanism unrelated to exaggerated inflammation or coagulation [36].

Possibly, the initial glucose level is a surrogate for a more profound stress hormone response in most afflicted patients [37]. Although the pathophysiology must be multimodal, the plausible mechanisms of this link involve toxicity, vasoconstriction and ischemia [32]. Hyperglycemia is a result of relative insulin deficiency, which is associated with increased lipolysis and excess circulating free fatty acids that are toxic to ischemic tissues [38]. Moreover, acute hyperglycemia diminishes endothelial nitric oxide, causing tissue hypoperfusion secondary to vasoconstriction. Nitric oxide is also used up by binding it to superoxide radicals generated by immune cells. During the binding, they generate peroxynitrites, which enhance platelet aggregation, thrombotic events and diminish mitochondrial function [39].

CK, another independent ACS predictor included in the risk score, is found in the cytosol of energy-consuming cells. Since there are different isoenzymes, CK is not to be viewed as a heart-specific enzyme and is no longer included in the diagnosis of heart attack. Currently, only troponin presents a specific cardiac enzyme used for the diagnosis of myocardial infarction in patients manifesting with appropriate clinical and/or electrocardiographic signs [40,41]. Our study was targeted at the early recognition of individuals prone to ACS (i.e., prior to the occurrence of myocardial ischemia). Therefore, the identification of such an ACS marker among routine laboratory parameters underlines the value of the proposed risk score as an optimal early screening tool. This finding enables risk stratification before the ACS event when the specific cardiac enzymes are thus far of limited practical relevance.

Notably, in our study, troponin levels routinely assessed at admission showed no predictive association with the risk of developing ACS. Furthermore, all ACS cases emerged during the 14-day intensive care monitoring period rather than within the initial 24 h post-admission. This timing suggests that ACS in our cohort likely had a multifactorial origin, arising from a mix of both preexisting cardiac conditions, such as coronary artery disease, and SAH-related catecholamine-induced myocardial stress. Therefore, admission troponin was not included in the final score.

If the patient is classified as a high-risk case after an assessment with the ACS score, it can be suggested to implement several preventive measures to reduce the risk of ACS in this subpopulation. These measures include more frequent monitoring of ECG and cardiac enzymes, a lower threshold for treating anemia, careful management of blood pressure and heart rate fluctuations as well as stress reduction strategies [42]. Provided the aneurysm has been treated, in select cases after a consultation with cardiology, prophylactic anticoagulation may be considered [43]. These measures aim to detect early signs of cardiac ischemia, maintain adequate oxygen supply to the myocardium, and minimize triggers that could precipitate an ACS event [44]. By implementing these preventive strategies, healthcare providers in the ICU can potentially reduce the incidence and severity of the impeding ACS.

The connection between GGT and ACS has been proven primarily for specific subgroups such as patients aged under 60 years [45,46]. Moreover, there is some evidence of a correlation between elevated hepatic enzymes and the severity of the ACS. Rajan et al. reported that raised GGT was significantly associated with major adverse cardiac events and in-hospital adverse outcomes, such as ventricular arrhythmia, heart failure and recurrent angina [47]. Regarding other hepatic enzymes, Jasiewicz and colleagues have prospectively assessed transaminase levels in patients with ACS. The majority of patients with more extensive myocardial injury presented high concentrations of GOT. Moreover, approximately 93% of patients with a large amount of infarcted myocardium presented GOT concentrations of over three times the upper limit of normal [48]. Studies on the role of GGT in aSAH patients were so far not published yet.

Dysnatremia is a common complication in patients with aSAH, most commonly as hyponatremia [49]. The current literature proposes that hyponatremia is diagnosed and treated during the course of the disease but is not associated with a worse neurological outcome or increased vasospasm [50,51]. Although serum sodium is present in some studies in connection with aSAH, it has not yet been evaluated with regard to the risk of ACS in aSAH patients. This circumstance urges the confirmation of this finding and other novel findings of ours in external aSAH populations.

In summary, the proposed risk score for ACS prediction is based on markers that are routinely assessed on admission in every aSAH patient. Since these parameters are available within one hour of hospitalization, the score can preemptively identify aSAH patients at increased risk of ACS as early as admission. Therefore, an ACS risk assessment with this score allows a preselection of cases at risk who could probably profit from a closer diagnostic surveillance of cardiac function for the early identification and prevention of ACS events.

## 5. Limitations

This study is retrospective in nature, which comes with typical limitations of such a design. Although based on a prospectively enrolling electronic database, the data completeness and accuracy are not as high as those in entirely prospective studies. Additionally, the findings are derived from data of a single hospital with internal treatment protocols. Given that ACS is a rare occurrence in cases of aSAH, the low number of ACS events may introduce a risk of overfitting the prediction model. Lastly, the novel ECG repolarization parameters, such as Tp-Te interval [52], Tpeak-Tend/QT ratio, Tpeak-Tend/QTc ratio [53], etc., were not included in the prediction model.

Due to this study’s single-center design and the infrequent occurrence of ACS in aSAH patients, a multicenter study with external validation is needed to evaluate the diagnostic accuracy of the ACS risk score presented here.

## 6. Conclusions

ACS is a rare, but severe, complication in aSAH patients and results in a significantly worse neurological outcome. We identified five independent weighted predictive factors for ACS in the context of aSAH: patient age (>55 years; 1 point), serum sodium (<142 mmol/L; 3 points), blood glucose (≥170 mg/dL; 2 points), serum CK (≥255 U/L; 3 points) and GGT (≥36 U/L; 1 point). Based on this score, patients with an increased risk of ACS could be identified with statistical reliability early in their treatment. External validation of the score is mandatory.

## Figures and Tables

**Table 1:** Patient baseline characteristics of the final aSAH cohort.

Parameter	Number of Cases, *n*	Percentage, %
Age over 55 years	373	43.6%
Sex (female)	580	67.8%
Ethnicity (Caucasian)	815	95.3%
Arterial hypertension present	591	69.2%
Smoking history	256	29.9%
Alcohol history	63	7.5%
Drug history	17	2.0%
Hypercholesterolemia	71	8.4%
Hypothyroidism	96	11.2%
Hyperthyroidism	10	1.2%
Hyperuricemia	21	2.5%
Pre-existing cardiac disease *	89	10.5%
Diabetes mellitus	47	5.5%
Chronic pain with NSAID treatment	55	6.5%
Beta-blocker treatment	131	15.6%
Calcium-antagonist treatment	80	9.5%
ACE-inhibitor treatment	152	18.1%
AT1-antagonist treatment	50	5.9%
Any anticoagulant pre-medication	72	8.4%
Aspirin pre-medication	60	7.0%
WFNS scale, grade = 4–5	354	41.4%
Fisher scale, grade = 3–4	654	85.8%
Intraventricular hemorrhage	386	45.5%
Intracerebral hemorrhage	251	29.4%
Treatment modality (clipping)	497	60.9%

Abbreviations: NSAID—non-steroidal anti-inflammatory drug; ACE—angiotensin-converting enzyme; AT1—angiotensin II type 1; WFNS—World Federation of Neurosurgical Societies; * We count coronary heart disease, myocardial damage and cardiac arrhythmias among the pre-existing cardiac diseases.

**Table 2:** Univariate analysis of ACS predictors.

Parameter	OR (95%-CI)	*p*-Value
Age > 55 years	2.66 (1.18–6.00)	0.017
Sex (female)	0.58 (0.26–1.26)	0.208
Ethnicity (Caucasian)	0.97 (0.95–0.98)	0.632
Arterial hypertension	1.57 (0.62–3.95)	0.401
Smoking history	1.17 (0.52–2.65)	0.674
Alcohol history	1.03 (0.23–4.48)	1.000
Drug history	0.96 (0.95–0.98)	1.000
Obesity (BMI > 25)	1.05 (0.24–4.56)	1.000
Hypercholesterolemia	2.04 (0.68–6.09)	0.266
Hypothyroidism history	0.29 (0.03–2.21)	0.349
Hyperthyroidism history	0.96 (0.95–0.97)	1.000
Hyperuricemia	3.5 (0.77–15.92)	0.134
Pre-existing cardiac disease *	1.57 (0.52–4.66)	0.342
Diabetes	2.32 (0.67–8.02)	0.168
Familial Intracranial Aneurysm	0.97 (0.96–0.98)	1.000
Chronic NSAID treatment	0.57 (0.08–4.26)	1.000
Beta-blocker treatment	1.65 (0.65–4.21)	0.274
Calcium-antagonist treatment	1.25 (0.36–4.26)	0.730
ACE-inhibitor treatment	2.07 (0.88–4.86)	0.115
AT1-antagonist treatment	0.62 (0.08–4.71)	1.000
Fisher score = 3–4	1.27 (0.37–4.32)	1.000
Present intraventricular hemorrhage	2.08 (0.94–4.61)	0.077
Graeb score ≥ 5	2.56 (1.15–5.72)	0.025
Intracerebral hemorrhage	1.01 (0.43–2.33)	1.000
WFNS score = 4–5	2.47 (1.12–5.47)	0.028
Treatment modality (clipping)	1.11 (0.48–2.54)	0.833
Admission ICP value > 20 mmHg	1.41 (0.65–3.04)	0.426
Aneurysm rebleeding	2.12 (0.61–7.31)	0.197
Acute hydrocephalus	1.51 (0.6–3.79)	0.522
Decompressive craniectomy	1.23 (0.53–2.87)	0.654
Primary decompressive craniectomy	1.01 (0.38–2.71)	1.000
Admission leukocytosis ≥ 11.0 × 109/L	2.81 (1.05–7.47)	0.030
Admission body temperature ≥ 38.0 °C	2.42 (1.07–5.48)	0.038
Admission sodium < 142 mmol/L	11.44 (1.53–85.44)	0.001
Admission chloride ≥ 106 mmol/L	0.47 (0.2–1.09)	0.105
Admission glucose ≥ 170 mg/dL	6.55 (2.47–17.4)	<0.0001
Admission blood urea < 17 mg/dL	0.46 (0.19–1.07)	0.091
Admission creatine kinase ≥255 U/L	5.85 (2.47–13.82)	<0.0001
Admission GOT ≥30 U/L	4.29 (1.79–10.27)	0.001
Admission GPT ≥26 U/L	2.99 (1.25–7.17)	0.015
Admission GGT ≥36 U/L	3.89 (1.63–9.33)	0.003
Admission LDH ≥259 U/L	4.99 (2.14–11.64)	<0.001

Abbreviations: *—We count coronary heart disease, myocardial damage and cardiac arrhythmias among the pre-existing cardiac diseases; OR—odds ratio; BMI—body mass index; NSAID—non-steroidal anti-inflammatory drug; ACE—angiotensin-converting enzyme; AT1—angiotensin II type 1; WFNS—World Federation of Neurosurgical Societies; ICP—intracranial pressure; GOT—glutamate-oxaloacetate-transaminase; GPT—glutamate-pyruvate-transaminase; GGT—gamma-glutamyltransferase; LDH—lactate dehydrogenase.

**Table 3:** The last step of the multivariable backward regression analysis of the ACS predictors with score weight and rounded weight (for all steps, see Supplementary Table S2 in Online Supplements).

Marker	aOR (95%-CI)	*p*-Value	Score Weight (Rounded)
Age > 55 years	3.36 (1.05–10.69)	0.040	1
Sodium (<142 mmol/L)	8.99 (1.11–72.97)	0.040	3
Glucose (≥170 mg/dL)	5.01 (1.67–14.99)	0.004	2
CK (≥255 U/L)	10.79 (3.42–34.03)	0.000	3
GGT (≥36 U/L)	3.19 (1.04–9.75)	0.042	1

Abbreviations: aOR—adjusted odds ratio; CI—confidence interval; CK—creatinine kinase; GGT—gamma-glutamyltransferase.

**Table 4:** Multivariate analysis for poor neurological outcome (mRS > 3) 6 months after aSAH occurrence of an ischemic stroke and intrahospital mortality.

Parameter	Unfavorable Outcome		Cerebral Infarction		In-Hospital Mortality	
	aOR (95%-CI)	*p*-Value	aOR (95%-CI)	*p*-Value	aOR (95%-CI)	*p*-Value
ACS	3.43 (1.15–10.27)	0.027	2.49 (0.94–6.60)	0.066	1.13 (0.39–3.23)	0.825
Age > 55 years	2.72 (1.88–3.92)	<0.001	1.32 (0.97–1.80)	0.078	1.92 (1.26–2.92)	0.002
WFNS 4–5	5.07 (3.48–7.38)	<0.001	2.50 (1.81–3.47)	<0.001	2.66 (1.71–4.14)	<0.001
Fisher 3–4	4.72 (2.03–10.98)	<0.001	1.53 (0.94–2.48)	0.084	13.03 (1.76–96.37)	0.012
Treatment (Clipping)	1.67 (1.16–2.40)	0.006	1.58 (1.15–2.15)	0.004	1.03 (0.67–1.56)	0.904

Abbreviations: aOR—adjusted odds ratio; CI—confidence interval; ACS—acute coronary syndrome; WFNS—World Federation of Neurological Surgeons.

## Data Availability

The data are not publicly available due to patient privacy protection, but anonymized data can be obtained from the corresponding author upon reasonable request.

## Supplementary Materials

The following supporting information can be downloaded at the following: https://www.mdpi.com/article/10.3390/medicina60111862/s1, Figure S1: Overview of the recruitment process of the study; Table S1: Univariate evaluation of continuous variables assessed as potential ACS predictors; Table S2: All ten steps of the multiple stepwise regression of the significant markers of the univariate analysis; Table S3: Relative risk of ACS concerning relevant clinical events during aSAH treatment.

## Abbreviations

Acute coronary syndrome	ACS
Adjusted odds ratio	aOR
Alkaline Phosphatase	AP
Angiotensin II type 1 receptor	AT1 receptor
Angiotensin-converting enzyme inhibitor	ACE inhibitor
Area under curve	AUC
Computed tomography	CT
Confidence interval	CI
Creatine kinase-myocardial band	CK-MB
Creatinine kinase	CK
Delayed cerebral ischemia	DCI
Digital subtraction angiography	DSA
Electrocardiography	ECG
Gamma-Glutamyltransferase	GGT
Glutamate-Oxaloacetate-Transaminase	GOT
Glutamate-Pyruvate-Transaminase	GPT
Intracranial pressure	ICP
Lactate Dehydrogenase	LDH
Modified Rankin scale	mRS
Non-Steroidal Anti-Inflammatory Drug	NSAID
N-terminal pro–B-type natriuretic peptide	NT-proBNP
Odds ratio	OR
Receiver operating characteristic	ROC
Subarachnoid hemorrhage	aSAH
World Federation of Neurological Surgeons	WFNS
