# Supplementary material for: Acute Coronary Syndrome After Aneurysmal Subarachnoid Hemorrhage: Incidence, Risk Factors and Impact on the Outcome
Source: Medicina (Kaunas). 2024 Nov 14;60(11):1862. doi: 10.3390/medicina60111862 (PMC11596182; doi:10.3390/medicina60111862)
Supplement: Supplementary file 1 [file medicina-60-01862-s001.zip › medicina-3218048-supplementary.pdf]

Supplementary Materials: Acute Coronary Syndrome After Aneurysmal Subarachnoid Hemorrhage: Incidence, Risk Factors and Impact on the Outcome

Džiugas Meška [1,*], Sebastian Schroer [1], Svenja Odensass [1], Meltem Gümüs [1], Christoph Rieß [1], Thiemo F. Dinger [1], Laurèl Rauschenbach [1], Adrian Engel [1], Marvin Darkwah Oppong [1], Yahya Ahmadipour [1], Yan Li [2], Philipp Dammann [1], Ulrich Sure [1] and Ramazan Jabbarli [1]

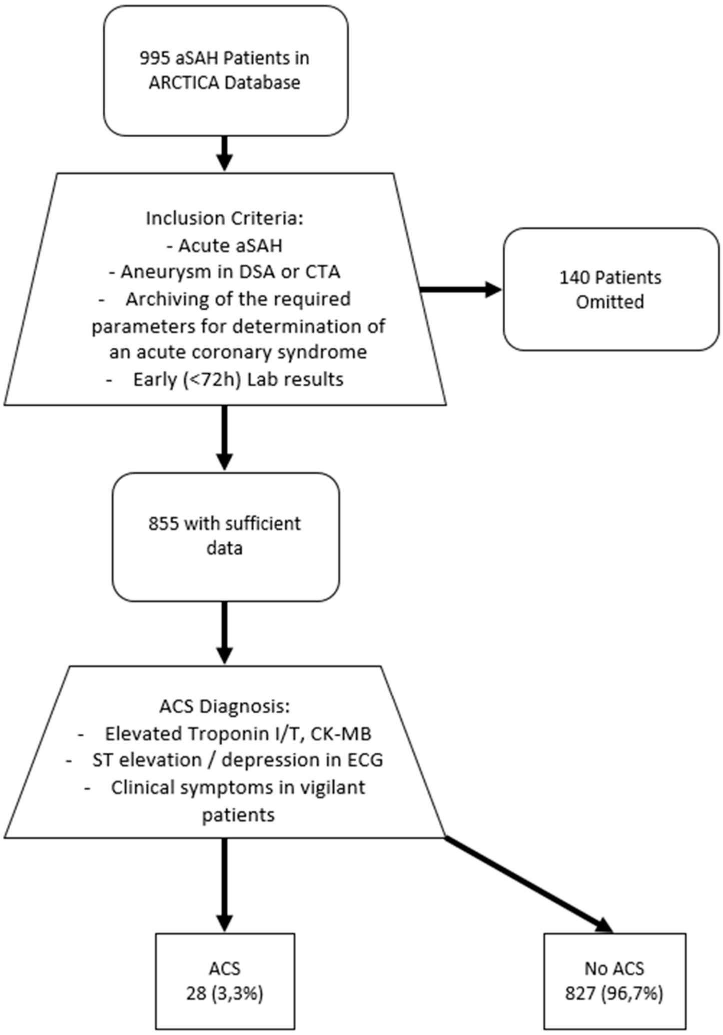

Figure S1: Overview of the recruitment process of the study. aSAH - aneurysmal subarachnoid hemorrhage. ACS – acute coronary syndrome. DSA – digital subtraction angiography. CTA – computer tomography angiography. ECG – electrocardiography.

Table S1: Univariate evaluation of continuous variables assessed as potential ACS predictors.

Parameter	Mean (standard deviation)		p-value
	No ACS	ACS present	
Age (Years)	54.65 (13.87)	61.46 (10.97)	0.005
Hijdra sum score	11.86 (8.95)	13.57 (8.97)	0.312
Original Graeb score	2.23 (3.3)	3.7 (4.05)	0.028
Aneurysm sack size (mm)	7.35 (4.77)	7.26 (4.76)	0.956
White Blood Cell count (*10^9/L)	13.05 (4.76)	14.93 (5.3)	0.065
Hemoglobin (g/dL)	12.53 (1.89)	12.68 (2.09)	0.695
C Reactive Protein (mg/L)	1.25 (2.37)	1.49 (1.91)	0.338
Interleukin-6 in liquor (pg/mL)	4183.58 (7558.82)	4318.88 (5220.62)	0.91
Cell count in liquor (/µL)	1711.72 (4869.4)	1392.61 (2032.97)	0.967
Troponin-I (pg/mL)	49.16 (407.48)	51.07 (209.39)	0.725
Min partial O2 pressure in blood gas (mmHg)	91.58 (26.04)	91.32 (29.95)	0.829
Min partial CO2 pressure in blood gas (mmHg)	30.73 (6.11)	31.14 (5.64)	0.667
Max partial CO2 pressure in blood gas (mmHg)	40.16 (7.93)	40.61 (7.39)	0.963

Max systolic blood pressure (mmHg)	163.41 (24.33)	171.75 (33.54)	0.144
Min systolic blood pressure (mmHg)	120.08 (20.03)	122.92 (24.88)	0.887
Difference between min and max systolic blood pressure (mmHg)	43.48 (26.06)	53.21 (44.89)	0.362
Mean arterial pressure (mmHg)	95.69 (10.78)	94.14 (12.02)	0.418
Max body temperature (°C)	37.16 (0.99)	37.52 (0.81)	0.051
Red blood cell count (*10^{12}/L)	4.36 (0.53)	4.36 (0.54)	0.892
Hematocrit (%)	38.78 (4.68)	38.4 (4.53)	0.523
Mean red blood cell volume (fL)	89.07 (5.84)	88.43 (6.45)	0.531
Mean red blood cell hemoglobin (pg)	30.46 (2.28)	30.35 (2.69)	0.899
Mean cellular hemoglobin concentration (g/dL)	34.21 (1.27)	34.26 (1.72)	0.862
Platelet count (*10^{9}/L)	237.96 (69.5)	247.08 (68.18)	0.131
Mean platelet volume (fL)	10.39 (0.89)	10.05 (0.95)	0.134
Sodium (mmol/L)	140.19 (3.77)	138.08 (4.1)	0.02
Potassium (mmol/L)	3.98 (0.44)	3.93 (0.5)	0.674
Chloride (mmol/L)	108.45 (5.62)	105.34 (6.11)	0.026

Calcium (mmol/L)	2.23 (0.16)	2.19 (0.12)	0.108
Glucose (mg/dL)	138.44 (49.72)	174.11 (44.94)	0.001
Creatinine (mg/dL)	0.93 (0.3)	0.94 (0.2)	0.641
Glomerular filtration rate (mL/min)	79.6 (21.07)	73.68 (11.87)	0.261
Urea concentration (mg/dL)	14.57 (7.66)	15.65 (3.98)	0.038
Creatin-Kinase (U/L)	221.53 (980.49)	595.26 (1607.7)	0.002
Bilirubin (mg/dL)	0.59 (0.34)	0.66 (0.47)	0.578
Direct bilirubin (mg/dL)	0.24 (0.26)	0.5 (0.5)	0.188
GOT (U/L)	33.16 (36.13)	49.82 (55.37)	0.001
GPT (U/L)	30.24 (33.9)	40.65 (29.27)	0.003
GGT (U/L)	46.07 (96.67)	44.26 (24.27)	0.008
LDH (U/L)	220.03 (73.98)	282.26 (108.28)	0.001
AP (U/L)	71.38 (34.3)	66.26 (23.7)	0.738
Amylase (U/L)	29.91 (26.78)	28.28 (16.11)	0.808
Total protein (g/dL)	6.49 (0.85)	6.49 (0.53)	0.724
Quick-Test (%)	95.7 (12.71)	89 (10.36)	0.214
Partial thromboplastin time (sec)	26.5 (10.77)	25.42 (1.85)	0.605
Prothrombin time (sec)	17.1 (1.31)	16.65 (0.73)	0.457

ACS – Acute coronary syndrome; GOT – Glutamate-Oxaloacetate-Transaminase; GPT – Glutamate-Pyruvate-Transaminase; GGT – Gamma-Glutamyltransferase; LDH – Lactate Dehydrogenase; AP – Alkaline phosphatase.

Table S2: All ten steps of the multiple stepwise regression of the significant markers of the univariate analysis.

Parameter		aOR	95% - CI		*p*-value
			lower	upper	
**Step 1**	Age over 55 years	3.38	0.88	13.04	0.077
	WFNS 4-5	0.61	0.16	2.31	0.468
	Intraventricular hemorrhage	0.79	0.18	3.39	0.751
	Graeb Score ≥ 5	2.15	0.45	10.29	0.339
	Temperature on admission (≥ 38.0°C)	0.85	0.22	3.29	0.816
	Leukocytosis on admission (≥ 11.0 $*10^9$/L)	1.49	0.41	5.46	0.545
	Sodium on admission (< 142 mmol/L)	10.68	1.26	90.32	0.030
	Glucose on admission (≥ 170 mg/dL)	5.77	1.62	20.56	0.007
	Blood urea nitrogen on admission (< 17 mol/L)	1.20	0.35	4.14	0.770
	CK on admission (≥ 255 U/L)	11.35	2.96	43.54	0.000
	GOT on admission (≥ 30 U/L)	1.36	0.25	7.47	0.724
	GPT on admission (≥ 26 U/L)	0.72	0.15	3.54	0.684
	GGT on admission (≥ 36 U/L)	3.25	0.85	12.43	0.085
	LDH on admission (≥ 259 U/L)	1.07	0.24	4.81	0.929

**Step 2**	Age over 55 years	3.39	0.88	13.06	0.075
	WFNS 4-5	0.62	0.17	2.27	0.469
	Intraventricular hemorrhage	0.80	0.19	3.38	0.757
	Graeb Score ≥ 5	2.13	0.45	10.07	0.341
	Temperature on admission (≥ 38.0°C)	0.85	0.22	3.26	0.811
	Leukocytosis on admission (≥ 11.0 *10^9/L)	1.50	0.41	5.45	0.537
	Sodium on admission (< 142 mmol/L)	10.6 6	1.26	90.26	0.030
	Glucose on admission (≥ 170 mg/dL)	5.75	1.62	20.44	0.007
	Blood urea nitrogen on admission (< 17 mol/L)	1.19	0.35	4.08	0.776
	CK on admission (≥ 255 U/L)	11.5 3	3.14	42.34	0.000
	GOT on admission (≥ 30 U/L)	1.40	0.29	6.73	0.674
	GPT on admission (≥ 26 U/L)	0.72	0.15	3.54	0.683
	GGT on admission (≥ 36 U/L)	3.25	0.85	12.42	0.085
**Step 3**	Age over 55 years	3.42	0.89	13.14	0.074
	WFNS 4-5	0.61	0.17	2.20	0.447
	Intraventricular hemorrhage	0.79	0.19	3.34	0.745
	Graeb Score ≥ 5	2.16	0.46	10.21	0.333

	Leukocytosis on admission (≥ 11.0 *10⁹/L)	1.50	0.41	5.41	0.537
	Sodium on admission (< 142 mmol/L)	10.47	1.25	87.95	0.031
	Glucose on admission (≥ 170 mg/dL)	5.60	1.61	19.45	0.007
	Blood urea nitrogen on admission (< 17 mol/L)	1.19	0.35	4.07	0.777
	CK on admission (≥ 255 U/L)	11.23	3.13	40.31	0.000
	GOT on admission (≥ 30 U/L)	1.43	0.30	6.83	0.654
	GPT on admission (≥ 26 U/L)	0.72	0.15	3.57	0.691
	GGT on admission (≥ 36 U/L)	3.15	0.84	11.74	0.087
**Step 4**	Age over 55 years	3.15	0.93	10.69	0.066
	WFNS 4-5	0.61	0.17	2.22	0.456
	Intraventricular hemorrhage	0.78	0.18	3.30	0.735
	Graeb Score ≥ 5	2.21	0.47	10.35	0.314
	Leukocytosis on admission (≥ 11.0 *10⁹/L)	1.47	0.41	5.25	0.556
	Sodium on admission (< 142 mmol/L)	10.63	1.26	89.46	0.030
	Glucose on admission (≥ 170 mg/dL)	5.55	1.60	19.19	0.007
	CK on admission (≥ 255 U/L)	11.05	3.10	39.39	0.000

	GOT on admission (≥ 30 U/L)	1.42	0.30	6.75	0.663
	GPT on admission (≥ 26 U/L)	0.72	0.15	3.57	0.692
	GGT on admission (≥ 36 U/L)	3.16	0.85	11.78	0.086
**Step 5**	Age over 55 years	3.12	0.92	10.60	0.067
	WFNS 4-5	0.60	0.17	2.16	0.435
	Graeb Score ≥ 5	1.92	0.53	7.02	0.322
	Leukocytosis on admission (≥ 11.0 *10^9/L)	1.45	0.41	5.13	0.568
	Sodium on admission (< 142 mmol/L)	10.50	1.25	88.26	0.030
	Glucose on admission (≥ 170 mg/dL)	5.47	1.59	18.83	0.007
	CK on admission (≥ 255 U/L)	11.08	3.11	39.47	0.000
	GOT on admission (≥ 30 U/L)	1.40	0.30	6.61	0.670
	GPT on admission (≥ 26 U/L)	0.71	0.15	3.45	0.668
	GGT on admission (≥ 36 U/L)	3.20	0.86	11.89	0.083
**Step 6**	Age over 55 years	3.12	0.92	10.56	0.068
	WFNS 4-5	0.64	0.18	2.24	0.485
	Graeb Score ≥ 5	2.08	0.59	7.27	0.252
	Leukocytosis on admission (≥ 11.0 *10^9/L)	1.41	0.40	4.97	0.592
	Sodium on admission (< 142 mmol/L)	10.53	1.25	89.03	0.031

	Glucose on admission (≥ 170 mg/dL)	5.21	1.55	17.54	0.008
	CK on admission (≥ 255 U/L)	11.7<br>1	3.36	40.75	0.000
	GPT on admission (≥ 26 U/L)	0.86	0.24	3.12	0.819
	GGT on admission (≥ 36 U/L)	3.29	0.90	12.09	0.072
**Step 7**	Age over 55 years	3.07	0.91	10.28	0.069
	WFNS 4-5	0.64	0.18	2.24	0.487
	Graeb Score ≥ 5	2.05	0.59	7.11	0.258
	Leukocytosis on admission (≥ 11.0 *10^9/L)	1.41	0.40	4.99	0.590
	Sodium on admission (< 142 mmol/L)	10.3<br>9	1.24	87.11	0.031
	Glucose on admission (≥ 170 mg/dL)	5.19	1.54	17.49	0.008
	CK on admission (≥ 255 U/L)	11.2<br>3	3.41	36.97	0.000
	GGT on admission (≥ 36 U/L)	3.09	0.95	9.97	0.060
**Step 8**	Age over 55 years	2.85	0.88	9.24	0.082
	WFNS 4-5	0.66	0.19	2.28	0.508
	Graeb Score ≥ 5	2.14	0.62	7.37	0.227
	Sodium on admission (< 142 mmol/L)	10.0<br>2	1.20	83.39	0.033
	Glucose on admission (≥ 170 mg/dL)	5.07	1.51	17.05	0.009

	CK on admission (≥ 255 U/L)	11.9 5	3.69	38.72	0.000
	GGT on admission (≥ 36 U/L)	2.99	0.93	9.63	0.066
**Step 9**	Age over 55 years	2.88	0.89	9.32	0.078
	Graeb Score ≥ 5	1.92	0.58	6.30	0.283
	Sodium on admission (< 142 mmol/L)	9.51	1.16	78.02	0.036
	Glucose on admission (≥ 170 mg/dL)	4.42	1.42	13.69	0.010
	CK on admission (≥ 255 U/L)	11.0 3	3.50	34.75	0.000
	GGT on admission (≥ 36 U/L)	3.30	1.06	10.24	0.039
**Step 10**	Age over 55 years	3.36	1.05	10.69	0.040
	Sodium on admission (< 142 mmol/L)	8.99	1.11	72.97	0.040
	Glucose on admission (≥ 170 mg/dL)	5.01	1.67	14.99	0.004
	CK on admission (≥ 255 U/L)	10.7 9	3.42	34.03	0.000
	GGT on admission (≥ 36 U/L)	3.19	1.04	9.75	0.042

aOR – Adjusted odds ratio; CI – Confidence interval; WFNS – World Federation of Neurosurgical Societies; CK – Creatinine kinase; GOT – Glutamate-Oxaloacetate-Transaminase; GPT – Glutamate-Pyruvate-Transaminase; GGT – Gamma-Glutamyltransferase; LDH – Lactate Dehydrogenase.

Table S3: Relative risk of ACS concerning relevant clinical events during aSAH treatment.

Clinical event	OR (95 %-CI)	p-value
Persistent ICP elevation requiring any (conservative/surgical) treatment	1.16 (0.54 - 2.51)	0.690
Secondary decompressive craniectomy due to ICP increase	1.41 (0.41 - 4.83)	0.470
Symptomatic cerebral vasospasm requiring endovascular treatment	0.92 (0.36 - 2.31)	1.000

OR – Odds ratio; CI – Confidence interval; ICP – Intracranial pressure.
